# Grooming-Related Concerns Among Companion Animals: Preliminary Data on an Overlooked Topic and Considerations for Animals' Access to Health-Related Services

**DOI:** 10.3389/fvets.2022.827348

**Published:** 2022-02-24

**Authors:** Shelby E. McDonald, Jessica Sweeney, Laura Niestat, Colleen Doherty

**Affiliations:** ^1^Department of Strategy and Research, American Society for the Prevention of Cruelty to Animals, New York, NY, United States; ^2^Community Engagement Program, Department of Humane Law Enforcement, American Society for the Prevention of Cruelty to Animals, New York, NY, United States; ^3^Veterinary Forensic Sciences, Department of Humane Law Enforcement, American Society for the Prevention of Cruelty to Animals, New York, NY, United States

**Keywords:** grooming, matting, companion animals, access to care, pet owners, animal welfare, animal cruelty

## Abstract

Grooming is an essential health maintenance activity that is fundamental to the welfare of many companion animals. Despite the potentially serious consequences of inadequate grooming for pets and their caregivers, few studies have examined the role of access to pet grooming services and supplies in promoting and maintaining companion animal health and welfare. The goal of this paper was 2-fold: (1) To provide preliminary findings demonstrating the scope of grooming and matting concerns among animals served by a large, non-profit animal welfare organization and (2) to provide a call for research to guide effective prevention of and responses to grooming-related omissions of care. We retrospectively extracted data from five American Society for the Prevention of Cruelty to Animals (ASPCA) programs serving the New York City area: ASPCA Animal Hospital (AAH), Community Medicine (CM), One ASPCA Fund, ASPCA-NYPD (New York City Police Department) Partnership, and the Community Engagement (CE) Program. The prevalence of grooming–related concerns was relatively consistent across all three veterinary service programs (AAH: 6%; CM: 4%; One ASPCA Fund: 6%). Thirteen percent of the ASPCA-NYPD Partnership's cruelty cases involved general hair matting concerns and/or strangulating hair mat wounds (93% were long-haired dog breed types). Five percent of CE cases received grooming-related supplies to support pet caregivers' in-home grooming capabilities. Our findings underscore the need to understand the scope of grooming-related concerns among animals served by veterinarians and other community programs to improve animals' access to health-related services.

## Introduction

Pet grooming is a health maintenance activity that is fundamental to the welfare of companion animals. Most companion animals require some degree of grooming, which can include basic hygiene care such as brushing, clipping, and trimming hair, bathing, cleaning the ears, and trimming claws. Inadequate grooming can lead to pain and discomfort for the animal and other threats to animal health and wellbeing. For example, when animals' claws are not adequately trimmed, they may alter the gait of the animal and make walking uncomfortable or challenging. In extreme cases, the claws may grow in a circular pattern and penetrate the paw pads on the underside of the feet causing painful wounds. Overgrown claws can alter the normal anatomic position and function of the feet (1). Additionally, some companion animals, such as long-haired dog breeds or mixes (e.g., Maltese, Shih Tzu, and Poodle), are particularly vulnerable to severe hair matting (2). Chronically matted hair can contribute to and cause medical conditions such as skin irritation and infection, recurrent or chronic ear and ocular infections and disease, anal soiling and obstruction, fecal constipation and impaction, urine scalding, and parasitic infestations (1–4). In some cases, chronically matted hair can encircle the lower limb(s) and constrict blood flow and lymphatic drainage resulting in soft tissue death, bone injury, and potentially amputation of the affected limb (2).

There are diverse reasons why a pet owner may not maintain their pet's grooming needs, some of which may be unintentional (e.g., lack of access to services, lack of knowledge regarding pet's grooming needs) and/or due to circumstances beyond their control [e.g., financial hardship, disability, mental illness, aging; (5, 6)]. Still, grooming-related omissions of care may meet legal definitions of animal neglect and have serious consequences for individuals who are unable or unwilling to provide adequate grooming-related care (7, 8). For example, if animal neglect is reported to law enforcement, these pet owners may face criminal charges. Yet these owners could be willing to provide grooming if barriers to care are addressed, making the need to understand the scope of grooming-related concerns among animals served by veterinarians, animal welfare organizations, and other community programs an important priority for advancing animal welfare and improving animals' access to health-related services.

### Pet Grooming in the U.S.

Nationally representative studies suggest that nearly 60% of U.S. households report having at least one pet, with dogs and cats being most prevalent (9). Lack of access to veterinary care, particularly for pets and people living in poverty, has gained increasing attention as an animal welfare issue in recent years (10). In this paper, we define access to veterinary care as the belief that, universally, companion animals should equitably receive compassionate, respectful, and considerate care that improves animal welfare, decreases suffering, and considers the needs of individual pets and family circumstances. Lack of access to veterinary care is a social problem that takes on many forms, including, but not limited to, pet owners' financial and physical barriers to care. Although grooming pets is essential to maintaining their health, access to grooming services and supplies, and access to related knowledge and professional advice, have typically been omitted from these conversations. During the COVID-19 pandemic, mainstream media highlighted debates as to the status of pet grooming services as an essential health-related service, with numerous media articles, news stories, and organized petition campaigns arguing that grooming services are essential to maintaining pets' health and wellbeing (11).

There is limited empirical data on pet grooming in the U.S. However, a recent report from the American Pet Products Association (12) indicates that in 2020, 81% of U.S. dog owners had groomed their pet in the past 12 months. Results of this survey suggest that at-home grooming is the most prevalent form of grooming (41%), followed by taking dogs to a full-service salon (30%), mobile grooming service (9%), retailer (8%), and self-service center (6%). On average, dogs were groomed professionally about four times during the past year, with the average number ranging from 3.2 (< $45K) to 4.6 ($125K+) across household income quartiles (i.e., < $45K, $45K-74.9K, $75K-124.9K, $125K+). Households in the two lowest income quartiles reported at-home grooming more often (i.e., < $45K: 44%, $45K-74.9K, 47%) than the higher income quartiles ($75K-124.9K: 38%, $125K+: 37%). Households in the two lowest income quartiles also reported using full-service salons less often (i.e., < $45K: 23%, $45K-74.9K: 28%) than the higher income quartiles (i.e., $75K-124.9K: 30%, $125K+: 36%). Although most dog owners engage in at-home grooming, ~23 and 57% of U.S. dog owners report that they do not own a brush or nail clippers, respectively, with low-income households reporting the lowest rates of owning these grooming tools (12).

Information regarding grooming practices of U.S. cat owners is limited despite evidence that brushing cats' hair is an important practice that serves to remove dead hair, aerate the skin, and disentangle knots that can cause pain and discomfort such as skin tightness and pruritus (an itching sensation), particularly in long-haired breeds (13). The APPA's recent report suggests that 32% of U.S. cat owners report that they do not have a brush or other grooming tool for their cat (12). Self-grooming is a typical feline behavior and, therefore, professional grooming is not essential for most cats, particularly short-haired breeds (14–16); however, recent data suggest that 17% of U.S. cat owners report that their pet has been groomed professionally in the past 12 months (12). Consistent with data on dog owners, utilization of professional grooming services among cat owners varies across income groupings, with only 13% of households with incomes under $25K reporting having had their cat groomed in the past 12 months vs. nearly a quarter of households with incomes over $125K (i.e., < $25K: 13%, $25K-44.9K: 14%, $45K-74.9K: 13%, $75K-124.9K: 19%, $125K+: 23%). To our knowledge, data regarding cat owners' methods of grooming (e.g., at-home vs. professional) and/or the type of grooming services accessed (e.g., mobile vs. full-service salon) are not available, nor are data on grooming practices by coat length or breed.

### Current Study

It is important that veterinary and animal welfare professionals and researchers consider access to grooming services and supplies when discussing animals' access to health-related services. This is of particular importance at the current time given that a recent nationally representative study suggests that one in five households acquired a new pet during the COVID-19 pandemic (13%). Moreover, “designer breeds” (e.g., Labradoodles), some of which require more intensive at-home and professional grooming for coat maintenance, are increasing in popularity (17–20). To advance this understudied area of animal welfare research, the goal of this paper is 2-fold. First, utilizing service data from a large, non-profit animal welfare organization, we provide preliminary data demonstrating the scope of need for grooming services across five service programs. Second, we provide a call to action for research on grooming-related omissions of care and outline future directions for research and practice in this area.

## Method

### Study Design and Sources of Data

This study was retrospective in design. All data reported on in the current paper were collected between 2018 and mid-2021 and reflect animals served in the New York City area. For the current study, we extracted data stored in electronic databases maintained by the ASPCA. Specifically, we examined data from five ASPCA program areas, which were stored in two databases (CiviCore and ImproMed). Each of the programs is described below.

### Program Descriptions

#### ASPCA Animal Hospital

AAH accepts cat and dog patients who require urgent or emergency care and belong to pet owners who are New York City residents and are experiencing financial hardships or constraints. Pet owners can schedule an appointment for their pets for veterinary care and/or be referred by other ASPCA programs in New York City and private veterinarians in the area. Services are either low or no-cost.

#### Community Medicine

CM provides high quality, high volume spay/neuter and primary veterinary care via the ASPCA's Community Veterinary Centers (CVCs) and mobile clinics in communities that experience barriers to veterinary care. Focus areas include the Bronx and Brooklyn. In addition to this work, CM, Community Engagement (described below), and the ASPCA Adoption Center collaborated to conduct a soft launch of grooming services at the CVCs and at weekly vaccine events in May 2021. Services available included basic grooming and nail trims, educational demonstrations for owners, and supplies (e.g., brushes and nail clippers). These appointments were exclusively for clients who had brought their pet(s) in for vaccines or preventive care. All services provided by CM are partially or fully subsidized. For grooming services rendered as part of the soft launch, we examined data from May 26, 2021 through July 28, 2021.

#### One ASPCA Fund

The One ASPCA Fund is a subsidy program for veterinary care facilitated by the ASPCA's Client and Member Support team to support and improve welfare for as many animals as possible. This program provides services for medical conditions that have a good prognosis and require short-term care. Clients qualify if they are referred by the ASPCA's Community Engagement or CM teams, the NYPD, or social service agencies (e.g., domestic violence shelters, food bank organizations).

#### ASPCA-NYPD (New York City Police Department) Partnership

The ASPCA partners with the NYPD to prevent and respond to animal cruelty in New York City. The NYPD responds to animal cruelty complaints, and the ASPCA directly cares for the animal victims by providing forensic evaluations, medical treatment, housing and placement, behavior assessments and treatment.

#### Community Engagement

The CE team works with New York City residents who lack access to vital care, services, and supplies for their pets. Often, these pet owners are referred to the team by the NYPD as cases that would benefit from receiving services rather than criminal justice action. CE provides families with resources to help them create and sustain a safe and healthy environment for their pet(s). The team also accepts referrals from social service and other allied agencies and conducts outreach throughout the community to increase awareness and access to the ASPCA's veterinary care and spay/neuter services, among other pet-related resources.

### Identification of Grooming-Related Appointments and Cases

To identify grooming-related appointments among our veterinary medicine programs, we examined all appointments with medically necessary grooming or nail trim noted in the animal's record. When a grooming service was not explicitly captured in the appointment data as a distinct field, we identified grooming-related appointments by reviewing the appointment reason as reported by the client, reviewing the DVM's appointment notes, and/or searching for phrases and words including medical grooming, sedated grooming, matting, matted, strangulation, unkempt, overgrown, ingrown, embedded, curled, curling, and other variations of these words and phrases. Cases and appointments involving ear cleaning and infections were not included because it was not possible to determine whether these appointments were related to grooming-related omissions of care. For data from the ASPCA-NYPD Partnership, cruelty cases that included any version of the words “matting” (e.g., mat, matted) or “strangulation” (e.g., strangulating wound) in the case description were included. A detailed overview of the procedures for identifying strangulating hair mats is provided in Watson and Niestat [(2), p. 28]. Approximately 15% of AAH appointments with medical grooming and/or nail trims involved animals that were served in association with an ASPCA-NYPD Partnership case. Therefore, some animals may be counted in both AAH and ASPCA-NYPD program estimates; however, we are unable to produce a precise estimate due to differences in data entry and storage across programs. For CE Program case data, cases that included any provision of grooming-related supplies to the client were included.

### Analysis

Data were exported to Microsoft Excel, which was used to produce descriptive statistics on grooming and matting-related cases and appointments for each program.

## Results

The number of cases and appointments per ASPCA program and the corresponding percentage involving grooming and/or hair matting concerns are provided in Table 1. Our data reflect more than 52,000 veterinary appointments from AAH, CM, and the One ASPCA Fund and 2,600 cases from the ASPCA-NYPD Partnership and the CE Program. Six percent of AAH's 2018–2021 appointments involved medically necessary grooming or nail trims. Four percent of appointments seen by CM veterinarians included grooming-related observations and/or service provision. Six percent of appointments scheduled via the *One* ASPCA Fund included grooming-related observations by the DVM and/or provision of grooming services.

**Table 1:** Number of appointments or cases per ASPCA program and the percentage involving grooming-related omissions of care and/or related services.

**Program**	**Percent of grooming-related appointments or cases**	**Species, services, and coat-type**	**Total appointments or cases**
AAH	6% (*n* = 1,230)	• 858 dogs, 372 cats • 421 medical grooming, 809 nail trim services ° 77% (*n* = 324) of medical grooming services were for long-haired breed types	19,327
ASPCA-NYPD Partnership	13% (*n* = 127)	• 97% (138) of the 142 animals involved in these cases were dogs ° 93% (*n* = 128) of dogs were identified as long-haired breed types	981
CE	5% (*n* = 79)	• 65 cases involved dogs only or both cats and dogs • 14 cases involved cats only • Breed-specific data not available	1,652
CM	4% (*n* = 1,266)	• 1,013 dogs ° 80% (*n* = 808) of dogs were identified as long-haired breed types • 252 cats ° 18% (*n* = 46) of cats were identified as long-haired breed types • 1 small mammal	31,047
Grooming at CVCs and Vaccine Events	100% (*n* = 204)	• 191 dogs ° 47% (*n* = 90) of dogs were identified as long-haired breed types ° 20% (*n* = 39) of dogs had no breed listed • 13 cats	204
OAF	6% (*n* = 119)	• 95 dogs ° 78% (*n* = 74) of dogs were identified as long-haired breed types • 23 cats • 1 turtle	2,154

*AAH, ASPCA Animal Hospital; CE, Community Engagement; CM, Community Medicine; CVC, Community Veterinary Center; OAF, One ASPCA Fund. Small, long-haired dog breeds and breed mixes represented in our data included: Bichon Frise, Brussels Griffon, Cavalier King Charles Spaniel, Cocker Spaniel, Coton de Tulear, Chinese Crested, Goldendoodle, Havanese, Lhasa Apso, Long-Haired Chihuahua, Maltese, Papillon, Pekingese, Pomeranian, Poodle, Scottish Terrier, Shih Tzu, Silky Terrier, Tibetan Terrier, West Highland Terrier, Wheaten Terrier, Yorkshire Terrier*.

Since 2018, more than 1 in 10 (13%) of the ASPCA-NYPD Partnership's cruelty cases have involved general hair matting concerns and/or concomitant strangulating wounds. Five percent of CE cases received grooming-related supplies to support in-home grooming capabilities (e.g., grooming kit, brushes, nail trimmers, shampoo). During the soft launch of the grooming services pilot program, 204 grooming appointments were provided at CVCs and weekly vaccine events; moreover, all available appointments were filled. At least 47% of these appointments served long-haired dog breeds or mixes (e.g., Maltese, Poodle, Shih Tzu, Pekingese); 20% of appointments did not capture the breed.

## Discussion

To our knowledge, this is the first paper to provide preliminary findings demonstrating the scope of grooming-related concerns among animals served by a large, non-profit animal welfare organization. We found that the prevalence of grooming–related concerns was relatively consistent across all three veterinary service programs (between 4 and 6%). Our results suggest that for many pet owners served by our animal welfare organization, especially by programs that aim to improve access to veterinary care through fully or partially subsidized services, earlier access to grooming care can help promote animal health and welfare. The level of need for medical grooming identified across these programs suggests that preventable issues, such as hair matting, likely diverts limited resources that could be better positioned for other veterinary care. For example, grooming is not on the menu of services provided by the ASPCA's veterinary programs. Thus, pets are typically brought in for another medical concern, and yet the animals' grooming need cannot be ignored and must be addressed by the veterinary staff at the same time. This could divert doctors' and staffs' time and resources away from treating other patients, which is of particular concern given the current veterinary staff shortage (21, 22).

Consistent with Watson and Niestat's (2) research, we found that small, long-haired dog breed types were overrepresented among appointments that involved grooming-related omissions of care, particularly medical grooming appointments. However, it is important to consider that the current study and Watson and Niestat's (2) relied on a sample of companion animals in the New York City area. It is possible that people in this region are more likely to own small dogs due to space limitations and restrictions associated with urban housing and therefore, these cases are overrepresented in our sample. We are not aware of any comparative data on rates of ownership by species and breed (e.g., long- vs. short-haired or single- vs. double-coated breeds) in New York City vs. nationally.

Our findings suggest that the provision of basic grooming services, facilitating clients' access to grooming services and supplies, and increasing clients' knowledge of their pet's grooming needs should be considered an important aspect of veterinary and animal welfare professionals' ability to provide a spectrum of care. Increasing clients' access to basic grooming may help to prevent more expensive and advanced care in the long-term and is particularly important for pet owners with financial limitations and those from underserved communities (23, 24). Access to veterinary care is essential so that companion animals can receive vaccinations (e.g., rabies, Bordetella) that are typically required for pets to receive professional grooming services. Moreover, veterinarians can play an important role in helping animals that are averse to grooming, such as making anxiolytic medications available to pet owners. When applicable, it is important that veterinarians assist clients in viewing the maintenance of pets' grooming needs as a viable and desirable choice for maintaining their pet's health and preventing negative health and behavioral outcomes. If grooming services and supplies are not available or cannot be accessed outside of veterinary settings, the consequences have the potential to become a burden on the veterinary community (e.g., medically necessary grooming), with potentially more serious implications for the non-profit veterinary community who likely encounter a disparate number of these cases.

This study also identified that the prevalence of grooming-related cases among animals served by the CE team was consistent with those served via the ASPCA's veterinary care programs (5%). This comparable rate is interesting to consider given that individuals who receive CE services and supports are often referred by social service and other allied agencies and by the ASPCA-NYPD Partnership in situations where non-criminal interventions can improve the health and safety of the animal. In contrast to the other ASPCA programs examined in this study, the rate of grooming-related concerns among animals served by the ASPCA-NYPD Partnership was notably higher and nearly double the rate found among the other programs at 13%. In addition, 97% of the ASPCA-NYPD Partnership's related cases involved dogs. The low rate of cases involving cats could be explained by prior evidence that cases of cruelty are more likely to receive prosecutorial attention if they involve dogs, despite other evidence that cats are the species most often involved in these cases (25–27). The higher rate of grooming-related omissions of care among the ASPCA-NYPD Partnership cases is not surprising given that these represent the most severe cases that have been recognized and reported by community members and service professionals (e.g., veterinary hospitals, animal welfare organizations) as animal cruelty or welfare concerns. Still, the percentage of cruelty cases involving matting or strangulation is likely underreported as only the case description was considered in the identification of cases included in our analysis. Moreover, omissions of care involving claws and other medical issues, such as myiasis, were not included in this estimate.

Findings from our examination of CE and ASPCA-NYPD Partnership cases suggest that programs aimed to improve access to health-related care among pet owners and those that provide direct human services would benefit from developing collaborative relationships with animal welfare organizations that can connect pet-owning clients with supplies and resources that help foster their ability to groom their pets. In addition, animal welfare organizations can be proactive in efforts to prevent grooming-related omissions of care. Examples of proactive efforts include providing trainings for allied professionals (e.g., child welfare workers, social services), educational materials for staff and clients (e.g., facts about pets' grooming needs, grooming demonstration videos, a list of community grooming services), and no- and low-cost services for their clients, as these populations likely face increased barriers to accessing health-related information and services for their pet. With more proactive support to meet pets' grooming needs, pet owners may intervene before hair matting or other identified medical problems significantly impact the animal's quality of life.

To prevent grooming-related omissions of care, it is important that the veterinary science and animal welfare fields consider how the social determinants of health that impact human health and wellbeing (e.g., transportation, neighborhood characteristics, income, education, discrimination) have a direct and indirect effect on pet owners' ability to groom their pets, particularly among marginalized communities. Pet owners may not be able to provide basic grooming care due to the conditions of the environment in which they live (28). For example, nationally representative data suggest that ~11.2 million dogs and 8.3 million cats in the U.S. live in under-resourced homes below the poverty line (29). In 2020, U.S. dog owners spent, on average, $197 on professional grooming at a salon, $161 on professional grooming from a mobile service, $47 on at-home grooming aids (e.g., brushes), and $40 on non-medicated shampoo and conditioner (12). For low-resourced individuals in low-resource economic conditions, pet grooming-related costs could lead to considerable financial strain and/or may not be a priority compared to providing the animal and other family members with basic needs such as food, water, and shelter. People may face additional barriers to pet grooming due to their lack of physical proximity to grooming services and supplies; commonly termed “animal resource deserts,” these communities often lack access to veterinarians, pet supply stores, and/or have little to no animal welfare infrastructure (30). Further, transportation to a professional grooming appointment, self-service salon, or pet supply store may be an additional barrier to grooming pets. Some people do not have access to a personal vehicle, pet-friendly public transportation, and/or equipment needed for traveling with pets (e.g., carrier, leash). For example, recent estimates suggest that 76 and 19% of U.S. dog and cat owners, respectively, do not have a crate or kennel for transportation of their pet (12). Research is needed to understand the independent, cumulative, and interactive effects of various forms of human adversity on pet owners' access to grooming services and supplies and ability to provide grooming-related care. We elaborate on opportunities for research in this area below, in our call for research.

It is common for private, for-profit veterinary practices to include non-medical services (i.e., grooming) within their business model as both an additional means of profit and as a convenience for pet owners. Our findings suggest that grooming services warrant consideration in the non-profit model as well, particularly in communities that lack access to pet care and/or have been historically excluded from vital pet care services. Given that many animal welfare organizations and shelters offer emergency sheltering and/or pet food pantries, it is conceivable that no- and low-cost pet grooming services could be added to expand the continuum of care and services provided for underserved animals and their caregivers. In addition to providing grooming services and supplies, programs that provide grooming demonstrations for pet owners and teach them to groom pets could be an effective way to prevent grooming-related omissions of care. Furthermore, it is possible that animal welfare organizations could partner with existing non-profit organizations that provide hygiene kits, access to mobile showers, and self-care resources for individuals who are housing insecure and expand the scope of services to provide animal-inclusive services that help to preserve the bonds between marginalized and underserved people and their pets.

Collectively, our results suggest that improving access to grooming services and supplies and improving caregivers' knowledge of their pets' grooming needs may improve the welfare of a significant number of companion animals served by programs that aim to improve underserved pet owners' access to veterinary care. Previous work has explored the concept of social determinants of animal health, a model that emphasizes health determinants that are important or unique to animals. For example, Card et al. discussed the intersection of human and animal social determinants of health and the importance of access to veterinary services in pet health equity (28). As social determinants of animal health continue to be discussed and envisioned by the veterinary field, we encourage veterinary and animal welfare scientists and professionals to consider and identify the significance of access to grooming services and supplies as a social determinant of animal health. Access to grooming is vital to the wellbeing of some companion animals, especially long-haired dogs and cats. Therefore, more equitable access to pet health services should include access to pet grooming and related supplies (28).

### Study Limitations

There are several limitations of our study that warrant consideration. First, our study was retrospective in design. Due to differences and changes in services, client eligibility parameters (e.g., income), and data entry and storage programs across ASPCA programs, our data points (i.e., timeline) vary slightly across programs. Second, we did not evaluate all aspects of grooming-related care, such as ear cleaning, or related medical issues such as myiasis. This was not possible due to inconsistencies in how programs collect these data. Third, it is important to note that there may be overlap between clients served through our veterinary services and those served by CE and the ASPCA-NYPD Partnership. Although we were able to identify the number of appointments, cases, and organizational resources serving animals who present with grooming-related concerns, we cannot identify the exact number of animals served as it is possible that some animals were double-counted due to how many times they were seen, both within and across programs. Fourth, our data reflect clients of a large non-profit organization in a large, urban city. Thus, our data may not generalize to other regions of the U.S. or animals served by organizations with fewer resources. Moreover, there is little marketing for the ASPCA's programs, and the services are limited, which likely makes the rates reported in this paper an underestimate of the scope of need. Finally, our datapoints span the time periods prior to, during, and after the height of the COVID-19 pandemic, which had major impacts on the operations of animal welfare organizations, veterinary practices, and law enforcement agencies.

### Call for Research

We conclude this paper with a call for research that aims to prevent and adequately respond to grooming-related omissions of care. First, to prevent grooming-related omissions of care and increase animals' access to health-related services, it is important to understand the scope of these concerns in general veterinary practice and in community and shelter medicine settings. We encourage other animal welfare professionals and researchers to examine and report on the scope of grooming-related concerns within animal care services. Such data will be critical to informing programs and policies that enhance grooming-related care. Furthermore, future work should aim to identify whether there are shared characteristics (e.g., neighborhood, poverty, age, culture) among pet owners whose animals are at risk for grooming-related omissions of care. Relatedly, understanding pet caregivers' beliefs and knowledge about grooming is essential to developing resources and programs that can successfully prevent and adequately respond to grooming-related omissions of care. Such information could help identify who may be most likely to benefit from knowledge and resources on grooming-related care and access to no- or low-cost services and/or how services and resources can be adapted to be culturally appropriate and responsive. Understanding discrepancies between veterinarian-identified grooming concerns and pet owners' awareness and concerns about these issues is also an important direction for future research.

As previously discussed, several individual and contextual factors likely serve as obstacles to adequate pet grooming, such as the owners' financial resources, proximity to grooming services, and access to pet-friendly transportation. Research is needed to understand these barriers and how they can be prevented, eliminated, and/or mitigated by non-profit animal welfare organizations, private veterinary clinics, and allied professionals. There is also a need to understand how pets' behavior impacts caregivers' ability to groom pets. For example, transporting pets to grooming appointments and/or attempting to groom them at home may be particularly difficult when animals have behavioral problems and behavioral conditions, such as aggression, anxiety, reactivity, and/or fear of being handled (31). Moreover, behavioral problems may be brought on or exacerbated by at-home and professional grooming. A recent evaluation of housed Maine Coon cats found that owners' grooming of cats (i.e., brushing) often elicited behaviors from the cat that are indicative of stress, such as aggressive behaviors, withdrawal, and facial discomfort, even when cats were habituated to brushing early in life (13). For novice pet owners, behavior problems and pets' reactions to grooming may present obstacles to meeting the pets' needs. Furthermore, prior research shows that pet owners may experience negative emotions (e.g., annoyance) and stress associated with their pets' behavioral problems; in turn, they may spend less time with pets (32, 33). If pet owners reduce the amount of time spent with pets due to the animal's behavioral problems, this may also impact the likelihood of consistently grooming the animal and retention of the pet. Future research should examine potential associations between pets' behaviors, grooming, and pet retention, as well as the role of the client-pet dynamic in grooming-related omissions of care.

Finally, the physical, mental, and cognitive health of pet owners and characteristics of their social relationships also have implications for the quality of care that animals receive and there is substantial need to understand how the psychological and physical health of pet owners may contribute to grooming-related animal welfare issues. For example, it may be unsafe for older pet owners and/or those with physical limitations or disabilities to groom pets at home. These owners may require assistance with traveling to grooming appointments and/or purchasing grooming supplies and performing grooming activities, such as bathing and nail trimming (34). Regarding mental and cognitive health, it is well-known that cognitive dysfunction, memory loss, depression, and trauma are associated with poor self-hygiene behaviors and neglect of child and adult dependents among adults (35–37). Adult humans' relationships with pets are often akin to a parental relationship with a child; therefore, these individual-level risk factors are important to consider in relation to inadequate grooming of pets (38–41). Lockwood found that 92% of respondents to a national survey of adult protective service workers had experienced animal neglect co-occurring with a client's inability to care for themselves (42). Household dysfunction (e.g., domestic violence, substance use) may also contribute to animal neglect (43–45). Indeed, there is some evidence that failure to groom pets' matted hair, seek veterinary care, and other forms of animal neglect are prevalent among households experiencing family violence (45, 46). Understanding how these individual and family-level factors impact pet owners' access to grooming services and supplies and ability to provide adequate grooming-related care is essential to establishing effective and sustainable programs. Therefore, we recommend research on the intersection of human and animal social determinants of health to guide future work in this area.

## Summary and Conclusion

Companion animals' grooming needs are an important aspect of their health-related care. Our findings provide preliminary evidence that improving access to grooming services and supplies and improving caregivers' knowledge of their pets' grooming needs is likely to improve the welfare of a significant number of companion animals. As few studies have examined pet owners' knowledge of their pet's grooming needs and/or barriers and facilitators of access to grooming services and supplies, we strongly advocate for continued research in this area. In addition, there is a great need for research aimed at establishing best practices for implementing programs that provide no- and low-cost grooming-related services and supplies for animals and their owners, particularly among underserved and low-resourced populations and communities. Consistent with prior work, our findings suggest that improved inter-agency and cross-services collaboration can help to ensure the health and welfare of multispecies families (5, 47).

## Data Availability Statement

The raw data supporting the conclusions of this article will be made available by the authors, without undue reservation.

## Author Contributions

SM and JS: conceptualization and methodology. JS: analysis. SM, JS, LN, and CD: writing, review, and editing. All authors contributed to the article and approved the submitted version.

## Funding

This study was funded by the ASPCA.

## Conflict of Interest

The authors declare that the research was conducted in the absence of any commercial or financial relationships that could be construed as a potential conflict of interest.

## Publisher's Note

All claims expressed in this article are solely those of the authors and do not necessarily represent those of their affiliated organizations, or those of the publisher, the editors and the reviewers. Any product that may be evaluated in this article, or claim that may be made by its manufacturer, is not guaranteed or endorsed by the publisher.
